# SFRP4 and CDX1 Are Predictive Genes for Extragastric Recurrence of Early Gastric Cancer after Curative Resection

**DOI:** 10.3390/jcm11113072

**Published:** 2022-05-29

**Authors:** Young Min Kim, In Gyu Kwon, Seung Ho Choi, Sung Hoon Noh, Jaeyoung Chun, Young Hoon Youn, Hyojin Park, Ji Hae Nahm, Jie-Hyun Kim, Yong-Min Huh, Eunji Jang

**Affiliations:** 1 Department of Internal Medicine, Gangnam Severance Hospital, Yonsei University College of Medicine, 211 Eonjuro, Seoul 06273, Korea; arsenalmdim@gmail.com (Y.M.K.); chunjmd@yuhs.ac (J.C.); dryoun@yuhs.ac (Y.H.Y.); hjpark21@yuhs.ac (H.P.); 2 Department of Surgery, Gangnam Severance Hospital, Yonsei University College of Medicine, 211 Eonjuro, Seoul 06273, Korea; surgeon@yuhs.ac (I.G.K.); choish@yuhs.ac (S.H.C.); sunghoonn@yuhs.ac (S.H.N.); 3 Department of Pathology, Gangnam Severance Hospital, Yonsei University College of Medicine, 211 Eonjuro, Seoul 06273, Korea; 4 Department of Radiology, Yonsei University, Seoul 03722, Korea; ymhuh@yuhs.ac; 5 YUHS-KRIBB Medical Convergence Research Institute, Yonsei University College of Medicine, 211 Eonjuro, Seoul 06273, Korea; 6 Brain Korea 21 Project for Medical Science, Yonsei University College of Medicine, 211 Eonjuro, Seoul 06273, Korea; 7 MediBio-Informatics Research Center, Novomics Co., Ltd., Seoul 07217, Korea; eunji.jang@novomics.com

**Keywords:** early gastric cancer, extragastric recurrence, single patient classifier genes, SFRP4, CDX1

## Abstract

Extragastric recurrence of early gastric cancer (EGC) after curative resection is rare, but prognosis has been poor in previous reports. Recently, single patient classifier (SPC) genes, such as secreted frizzled-related protein 4 (SFRP4) and caudal-type homeobox 1 (CDX1), were associated with prognosis and chemotherapy response in stage II–III gastric cancer. The aim of our study is, therefore, to elucidate predictive factors for extragastric recurrence of EGC after curative resection, including with the expression of SPC genes. We retrospectively reviewed electronic medical records of 1974 patients who underwent endoscopic or surgical curative resection for EGC. We analyzed clinicopathological characteristics to determine predictive factors for extragastric recurrence. Total RNA was extracted from formalin-fixed, paraffin-embedded (FFPE) tumor tissue and amplified by real-time reverse transcription polymerase chain reaction to evaluate expression of SPC genes. Overall incidences of extragastric recurrence were 0.9%. In multivariate analysis, submucosal invasion (odds ratio [OR] = 6.351, *p* = 0.032) and N3 staging (OR = 171.512, *p* = 0.012) were independent predictive factors for extragastric recurrence. Mean expression of SFRP4 in extragastric recurrence (−2.8 ± 1.3) was significantly higher than in the control group (−4.3 ± 1.6) (*p* = 0.047). Moreover, mean expression of CDX1 in extragastric recurrence (−4.6 ± 2.0) was significantly lower than in the control group (−2.4 ± 1.8) (*p* = 0.025). Submucosal invasion and metastasis of more than seven lymph nodes were independent predictive factors for extragastric recurrence. In addition, SFRP4 and CDX1 may be novel predictive markers for extragastric recurrence of EGC after curative resection.

## 1. Introduction

The current standard treatment for early gastric cancer (EGC) includes endoscopic and surgical resection depending on the clinical staging [1,2]. Regarding the prognosis of EGC, 5-year overall survival and disease-specific survival rates were approximately 92% and 99%, respectively [3,4]. Despite this excellent prognosis, patients who experienced recurrence can still show unfavorable outcomes, as evidenced by prior reports. One previous study reported that 1.4% of patients experienced recurrence after curative resection for EGC, and median survival of these patients was just 4.3 months [5]. Therefore, determining the predictive factors for recurrence is of particular importance. The recurrence rates of EGC after curative resection varied from 1.5% to 15% in other studies [6,7]. Moreover, extragastric recurrence is rare, with an incidence rate of around 1.0% [5,8,9]. As a result, studies demonstrating extragastric recurrence of EGC after curative resection have been scarce.

In the case of advanced gastric cancer, several studies have reported therapeutic strategies targeting human epidermal growth factor receptor 2 and the tumor microenvironment [10,11]. Moreover, third-line and salvage treatment have shown superior overall survival and progression-free survival to placebo or best supportive care in advanced or metastatic gastric cancer [12]. Recently, several studies have discussed predictors for prognosis and treatment response in gastric cancer [13,14,15,16,17]. Among these, one multicenter study introduced single patient classifier (SPC) genes into stage II–III resectable gastric cancer [17]. The SPC genes included secreted frizzled-related protein 4 (SFRP4), caudal-type homeobox 1 (CDX1), granzyme B (GZMB), and tryptophanyl-tRNA synthetase (WARS). Patient prognosis and prediction of chemotherapy response were evaluated according to the expression of the SPC genes at the mRNA level. The findings showed significant prognostic SPC (SFRP4, GZMB, and WARS) and predictive SPC (CDX1, GZMB, and WARS) expressions. Elevated expression of SFRP4 was associated with poor prognosis, and low expression of CDX1 was a predictor of a lack of benefit from chemotherapy.

Based on the aforementioned study, we hypothesized that there would be an association between the expression of SPC genes and EGC, especially in the case of extragastric recurrence. Hence, this study aimed to investigate the patterns and predictive factors for extragastric recurrence of EGC after curative resection, including with the expression of SPC genes.

## 2. Methods

### 2.1. Study Design and Population

**Figure 1:**
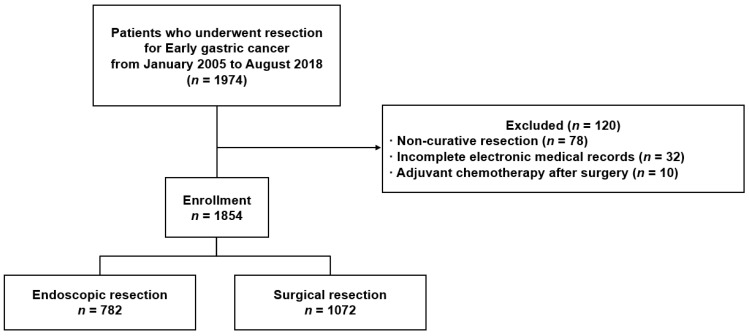
Flow chart of enrolled patients.

We retrospectively reviewed the electronic medical records of 1974 patients who underwent endoscopic or surgical resection for EGC at Gangnam Severance Hospital in Seoul, Korea between January 2005 and August 2018 (Figure 1). Only Korean patients over 18 years of age were included, and the exclusion criteria were as follows: (1) non-curative resection (*n* = 78), (2) incomplete electronic medical records (*n* = 32), and (3) adjuvant chemotherapy after surgical treatment (*n* = 10). As a result, 1854 patients were enrolled in this study. With regard to the method of resection, 782 patients (42.2%) underwent endoscopic resection (720 for endoscopic submucosal dissection and 62 for endoscopic mucosal resection) and 1072 patients (57.8%) underwent surgical resection (129 for total gastrectomy and 943 for subtotal gastrectomy).

The study protocol conformed to the ethical guidelines of the World Medical Association Declaration of Helsinki and was approved by the Institutional Review Board of Gangnam Severance Hospital (3-2019-0224). Informed consent was obtained for using existing resected tissue for gene expression analysis.

### 2.2. Data Collection

We collected information pertaining to patient demographics and lesion characteristics from electronic medical records. The collected demographic information included age, sex, and follow-up period. Lesion characteristics were divided into endoscopic and pathologic findings. Endoscopic findings included location, maximal diameter, multiplicity, and gross appearance of the lesions. Tumor location was divided into the upper-, middle-, and lower-third of the stomach. Gross appearance was divided into elevated, flat, and depressed types according to the classifications issued by the Japanese Research Society for Gastric Cancer [18]. In the case of lesions of a mixed gross type, these were considered to be prominent in appearance. Pathologic findings included histologic type, Lauren classification, and depth of invasion. Moreover, we evaluated lymph node (LN) status in the case of surgical resection. Histologic type was classified as either differentiated or undifferentiated, in accordance with the Japanese guidelines [19]. Well- and moderately differentiated tubular adenocarcinoma were categorized as differentiated types, while poorly differentiated tubular adenocarcinoma and signet ring cell adenocarcinoma were categorized as undifferentiated types. LN status was evaluated according to the 8th edition of the American Joint Committee on Cancer (AJCC) [20].

### 2.3. Definition of Recurrence

Recurrence was detected using the follow-up program, which included imaging study and upper endoscopy. Extragastric recurrences included locoregional, hematogenous, and peritoneal patterns. The sites of locoregional recurrence included regional LNs, such as perigastric, retropancreatic, gastrohepatic ligament, and para-aortic LNs. The sites of hematogenous recurrence were categorized as liver, lung, bone, adrenal gland, and distant LNs, such as supraclavicular LN.

The ‘no recurrence’ group was defined as patients who did not experience recurrence during the follow-up period. Moreover, we created a ‘control’ group for comparison with the extragastric recurrence group in terms of gene expression. For each patient included in the extragastric recurrence group, we included 2 patients in the control group. Therefore, the sample size of the control group was twice as large as that of the extragastric recurrence group. Other than the absence of recurrence during the follow-up period, the criteria for the control group were the same as those for the extragastric recurrence group and included sex, gross appearance, histologic type, Lauren classification, depth of invasion, and LN status in the case of surgical resection. Notably, there was no significant difference in LN status between the two groups. Supplementary Table S1 shows the result of statistical analysis for LN status between the extragastric recurrence and the control groups.

### 2.4. Definition of Curative Resection

Endoscopic curative resection was achieved when tumors were completely resected, and final pathological evaluation results met the curability criteria for absolute or expanded indications of endoscopic resection. Surgical curative resection refers to R0 resection, which indicates a microscopically margin-negative resection, in which no gross or microscopic tumor remains in the primary tumor bed.

### 2.5. Gene Expression Analysis

**Figure 2:**
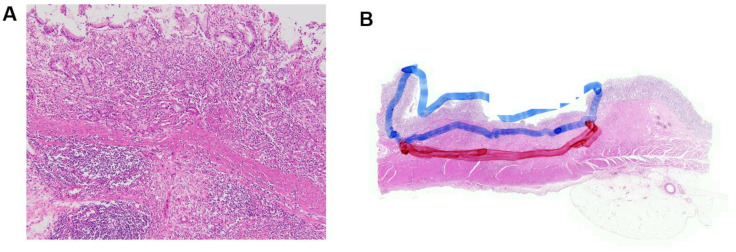
Representative gene expression analysis of a patient who received total gastrectomy for early gastric cancer. (**A**) Signet-ring carcinoma and submucosal invasion (×100). (**B**) Blue-colored circle in mucosal cancerous lesion and red-colored circle in submucosal cancerous lesion as observed on hematoxylin and eosin-stained slide. * Gene expression analysis results in this case; SFRP4 expression: −3.39 in mucosa and −2.71 in submucosa; CDX1 expression: −4.07 in mucosa and −4.94 in submucosa. SFRP4, secreted frizzled-related protein 4; CDX1, caudal-type homeobox 1.

To analyze the expression of candidate genes, namely, SFRP4 and CDX1, formalin-fixed, paraffin-embedded (FFPE) tumor tissues designated for use as endoscopic and surgical specimens were sliced into 4-µm-thick tissue sections. The representative cancer regions of the mucosa and/or submucosa were identified and marked on the hematoxylin and eosin (H&E) stained slides by a pathologist. A previous study by Minata et al. reported that the gene expressions of the tumor core and invading edge are different [21]. Therefore, in cases of submucosal cancer, we marked the cancerous regions of the mucosa and submucosa with different colors and performed gene analysis in these 2 regions separately (Figure 2).

Equal amounts of tumor tissue were obtained for RNA extraction by microdissection from 6–10 unstained paraffin sections, using the H&E slides for guidance. To quantify the RNA expression levels of the target genes, the nProfiler 1 Stomach Cancer Assay (Novomics, Seoul, Korea), based on the real-time reverse transcription polymerase chain reaction, was used.

We performed gene expression analysis in the extragastric recurrence and control groups, and the gene expression data for each case were recorded as numerical values. Since there is no established cut-off value for candidate genes in EGC, we did not include SPC genes as confounding factors in the multivariate analysis. Instead, we compared the results of gene expression analysis between the extragastric recurrence and the control groups, which were both matched in terms of clinicopathological factors.

### 2.6. Follow-Up Program

Regular follow-up was performed in the patients after curative resection. In the patients who underwent endoscopic resection, follow-up was conducted every 6 months during the first 3 years and once every year thereafter. The follow-up interval of the patients who underwent surgical resection varied according to the staging. As with the endoscopic resection group, patients with stage I EGC underwent follow-up tests every 6 months during the first 3 years and once every year thereafter. In the case of stage II EGC or above, patients were followed up every 3–6 months during the first year, every 6 months during the next 5 years, and once every year thereafter. At each follow-up visit, the patients underwent physical examination, upper endoscopy, and radiologic studies, including abdominal computed tomography (CT).

### 2.7. Statistical Analysis

Continuous variables were reported as mean ± standard deviation and were compared between groups using either *t*-test or Wilcoxon rank-sum test. Categorical variables were reported as numbers and percentages, and chi-squared test or Fisher’s exact test were used to compare them. We performed multivariate logistic regression analysis to identify the predictive factors for extragastric recurrence of EGC after curative resection. Statistical analysis was performed using SPSS version 25.0 (IBM Corp., Armonk, NY, USA). A 2-tailed *p*-value of <0.05 was considered statistically significant.

## 3. Results

### 3.1. Baseline Characteristics

Of the 1854 patients whose records were included, 1227 (66.2%) were men, and the mean age was 60.2 ± 11.8 years (Table 1). The average follow-up period was 46.0 ± 29.6 months. Approximately 67.7% of all lesions were located in the lower third of the stomach. The mean maximal diameter of the lesions was 23.8 ± 17.0 mm. Submucosal invasion was observed in 595 lesions (32.1%). Among the 1072 cases of surgical resection, 8.8% of the lesions showed LN metastasis (LNM).

### 3.2. Patterns of Extragastric Recurrence

In this study, 16 patients (0.9%) experienced extragastric recurrence (Table 2). The mean time to extragastric recurrence was 25.7 ± 15.6 months. With regard to the method of resection, a total of 1.1% of the surgical resections and 0.5% of the endoscopic resections resulted in extragastric recurrence. Among the aforementioned 16 patients, one experienced multiple site recurrence (LN, bone, and adrenal gland). The most common pattern of extragastric recurrence was hematogenous recurrence (10 cases), occurring in the liver, lung, bone, adrenal gland, and distant LNs. Locoregional recurrence (four cases) in regional LN was also reported, as was peritoneal recurrence (four cases). Among those who underwent endoscopic resection, locoregional recurrence in regional LN was the most common pattern of extragastric recurrence. Regarding surgical resection, hematogenous recurrence was the most common pattern of extragastric recurrence.

Additionally, intragastric recurrence, which is defined as a local recurrence with a positive margin, was observed in six patients (0.3%). Moreover, metachronous multiple gastric cancer was reported in 32 patients (1.7%).

### 3.3. Predictive Factors for Extragastric Recurrence

Upon univariate analysis, location, Lauren classification, depth of invasion, and LNM status showed a significant association with extragastric recurrence. In the multivariate analysis, submucosal invasion (odds ratio [OR] = 6.351, 95% confidence interval [CI]: 1.251–39.352, *p* = 0.032) and N3 staging, defined as the metastasis of more than seven LNs, (OR = 171.512, 95% CI: 4.359–7965.315, *p* = 0.012), were independent predictive factors for extragastric recurrence (Table 3).

### 3.4. The Result of Gene Expression Analysis

**Figure 3:**
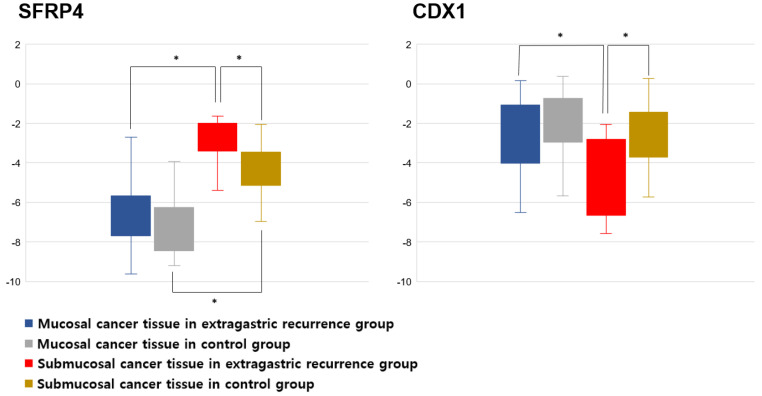
The result of gene expression analysis for SFRP4 and CDX1. SFRP4, secreted frizzled-related protein 4; CDX1, caudal-type homeobox 1. * *p* < 0.05.

Figure 3 shows the expression of SFRP4 and CDX1 in 16 extragastric recurrences and 32 control cases, respectively. The mean SFRP4 expression of submucosal cancer tissue in the extragastric recurrence group (−2.8 ± 1.3) was significantly higher than that in the control group (−4.3 ± 1.6) (*p* = 0.047). In both the extragastric recurrence group (−2.8 ± 1.3 vs. −6.6 ± 1.8, *p* < 0.001) and the control group (−4.3 ± 1.6 vs. −7.1 ± 1.5, *p* < 0.001), SFRP4 expression was significantly higher in the submucosal cancer tissue than that in the mucosal layer. The mean CDX1 expression in the submucosal cancer tissue of the extragastric recurrence group (−4.6 ± 2.0) was significantly lower than that in the control group (−2.4 ± 1.8) (*p* = 0.025). In the extragastric recurrence group, the expression of CDX1 in the submucosal cancer tissue was significantly lower than the expression observed in the mucosal layer (−2.4 ± 1.8 vs. −2.6 ± 2.0, *p* = 0.047). Among the 16 patients who experienced extragastric recurrence in our study, 10 received chemotherapy, one received radiotherapy for recurrence, and five were follow-up loss.

## 4. Discussion

Since extragastric recurrence of EGC after curative resection can be fatal, it is important to determine the predictive factors for extragastric recurrence. We found submucosal invasion and the presence of metastasis in seven or more LNs to be independent predictive factors for extragastric recurrence. Moreover, SFRP4 and CDX1 were found to be predictive markers for extragastric recurrence after curative resection.

Until now, studies regarding SPC genes have focused on advanced gastric cancer. Busuttil, R.A. et al. evaluated the role of SFRP4 in the prediction of tumor invasion and recurrence of advanced gastric cancer [22]. Moreover, another study elucidated the role of SPC as a prognostic marker of pT1N1 gastric cancer [23]. To the best of our knowledge, this study is the first to identify an SFRP4 and CDX1 as a predictive biomarker for extragastric recurrence of EGC.

In our study, the incidence of extragastric recurrence was 0.9%. The relative rarity of extragastric recurrence is in line with previous studies [5,6,7,8,9]. The mean time to extragastric recurrence was approximately 3 years. During this period, patients should be placed under strict surveillance, including imaging and endoscopic evaluations for the detection of recurrence. In our study, all 16 cases of extragastric recurrence were initially detected by abdominal CT. Regarding the overall recurrence pattern, hematogenous recurrence, including that in the liver, lung, bone, and adrenal gland, was the most common pattern of extragastric recurrence. This is in accordance with the findings of prior reports [24,25].

Several predictive factors for extragastric recurrence were identified, such as old age, male sex, elevated gross appearance, and LNM [9,25,26]. In our study, submucosal invasion (OR 6.351) and the presence of metastasis in more than seven LNs (OR 171.512) were significant predictive factors for extragastric recurrence of EGC after curative resection. This finding supports the notion that hematogenous recurrence is the most common pattern of extragastric recurrence in our study. Hematogenous recurrence is known to be associated with submucosal invasion of tumor cells and hence lymphovascular seeding [27,28]. Unlike previous reports, which only considered the negative or positive status of LNM, our study subdivided LNM into four categories as classified in the 8th edition of the AJCC. This distinction is a strength of our study. Previous studies reported that the number of harvested LNs was related to survival in gastric cancer [29]. Therefore, the thorough dissection of LNs is important for the evaluation of LNM as well as the prediction of extragastric recurrence.

Since one of the inclusion criteria was EGC, which shows a favorable prognosis, SFRP4 as stem-like module and CDX1 as intestinal epithelial module were candidate genes in our study. The major role of GZMB and WARS as immune modules was evaluation in terms of prognosis and chemotherapy response in stage II–III gastric cancer [17]. Therefore, these two genes were excluded for candidate genes in this study.

In our study, SFRP4 was found to be a positive predictive marker for extragastric recurrence of EGC after curative resection. SFRPs act as Wnt signaling modulators and were initially recognized for their tumor-suppressing properties in early studies [30,31]. However, a recent study showed that some subtypes of SFRPs, such as SFRP2 and SFRP4, tended to be overexpressed in cancer [32]. Regarding the association between SFRP4 and gastric cancer, the elevated expression of SFRP4 was significantly related to poor prognosis in stage II–III gastric cancer [17]. On the other hand, CDX1 is an intestine-specific transcription factor with an important role in the development of the intestines [33,34]. Previous studies have shown that CDX1 is associated with intestinal differentiation along the gastric carcinogenesis pathway [34]. One single center study reported that the expression of CDX was significantly associated with intestinal metaplasia, a premalignant lesion with known associations to gastric malignancy [35]. Moreover, CDX1 was found to be a predictive marker for chemotherapy response in stage II–III gastric cancer from the previous study [17].

The gene analysis revealed that the mean expression of CDX1 in patients with a partial response (−2.4 ± 0.5) was higher than that in patients of progressive disease (−7.4 ± 3.4). This finding was in accordance with the result of stage II–III gastric cancer. Additionally, we found CDX1 to be a negative predictive marker for the extragastric recurrence of EGC.

In a previous study, which analyzed SPC genes in stage II–III gastric cancer [17], only eight EGC patients (T1N2) were included. Among these eight patients, one patient expired due to cancer recurrence. Regarding SPC genes, this expired patient showed the highest expression of SFRP4 (−3.99) and the lowest expression of CDX1 (−9.68) among the eight patients. This tendency was consistent with the result of our study.

Our study aimed to determine the clinically useful factors for extragastric recurrence, including SPC genes. The expression of SFRP4 and CDX1 in the extragastric recurrence of EGC showed a significant difference when compared with the control group, especially in the submucosal invasive cancer cells. Hence, our findings confirm that SFRP4 and CDX1 may serve as novel predictive markers for the extragastric recurrence of EGC after curative resection. Moreover, when deciding how to approach gene analysis in the case of submucosal invasion, it appears that the expression of candidate genes is more significant in the submucosa. Therefore, it is better to obtain specimens from the submucosa rather than the mucosa.

This study has several strengths. First, our study evaluated the recurrence patterns and predictive factors for both endoscopic and surgical treatment of EGC. Although these methods are both common treatment modalities, most previous studies have assessed only one or the other. Second, to identify predictive factors, we analyzed not only clinicopathological features but also the result of gene expression using in vitro data. Most studies regarding the predictive factors of recurrence have mainly investigated patient characteristics, endoscopic findings, or pathological findings. Although these factors are important in the prediction of recurrence, gene expression is more closely associated with the pathogenesis of gastric carcinogenesis and recurrence. Third, our study provides the foundation for future research into the genetic aspects of gastric cancer. For instance, comparing the expression of candidate genes in cancerous lesions and recurrence sites will provide important data for the determination of clear mechanisms of extragastric recurrence.

There were some limitations in our study. First, this was a retrospective study with a relatively small sample size of patients with extragastric recurrence. Therefore, we did not establish the optimal cut-off value for SPC genes as in stage II–III gastric cancer [17] and include SPC genes in multivariate analysis. To overcome this limitation, we compared the expression of SPC genes between extragastric recurrence and the control group, which matched clinopathological factors such as the same depth of invasion and same LN status with extragastric recurrence cases. However, a prospective, multi-center study is warranted to establish the cut-off value and confirm the significance of our findings. Second, this study only enrolled members of the Asian population. Third, we did not perform immunohistochemistry staining to confirm protein level. There may be a concern regarding the reliability of RNA quantitation because total RNA was extracted from FFPE tumor tissues in this study. However, the real-time RT–PCR overcame this limitation by including the design of optimal primers and amplicons. A real-time RT–PCR-based predictive test using FFPE breast cancer tissues has been used in routine clinical practice [36]. Finally, the mechanisms linking the expression of SFRP4 and CDX1 to extragastric recurrence remain unclear. Our study only evaluated the association between extragastric recurrence and SPC genes. However, it is likely that these genes play a crucial role in modulating the signaling pathway and differentiation of epithelial cells. Further molecular studies should be performed to identify the precise mechanisms.

## 5. Conclusions

Hematogenous recurrence was the predominant pattern of extragastric recurrence. The mean time to extragastric recurrence was 25.7 ± 15.6 months, and close monitoring is required during this period. Submucosal invasion and the presence of metastasis in more than seven LNs were found to be independent predictive factors for extragastric recurrence. High levels of SFRP4 and low levels of CDX1 were significantly associated with extragastric recurrence. Therefore, the expression of SPC genes may serve as novel predictive markers for the extragastric recurrence of EGC after curative resection.

## Figures and Tables

**Table 1:** Baseline characteristics of the study population.

Characteristics	All Patients (*n* = 1854)
Study population	
Age (years, mean ± SD)	60.2 ± 11.8
Male (*n*, %)	1227 (66.2)
Follow-up period (month, mean ± SD)	46.0 ± 29.6
Endoscopic finding	
Location (*n*, %)	
Upper third	165 (8.9)
Middle third	434 (23.4)
Lower third	1255 (67.7)
Maximal diameter (mm, mean ± SD)	23.8 ± 17.0
Multiplicity (*n*, %)	
Single lesion	1776 (95.8)
Multiple lesion	78 (4.2)
Gross appearance (*n*, %)	
Elevated	368 (19.8)
Flat	712 (38.4)
Depressed	774 (41.7)
Pathologic finding	
Histologic type (*n*, %)	
Differentiated	1184 (63.9)
Undifferentiated	670 (36.1)
Lauren classification (*n*, %)	
Intestinal	1235 (66.6)
Diffuse	520 (28.0)
Mixed	99 (5.3)
Depth of invasion (*n*, %)	
Mucosa	1259 (67.9)
Submucosa	595 (32.1)
Lymph node status * (*n*, %)	
Negative	978/1072 (91.2)
Positive	94/1072 (8.8)

* Only evaluated in the case of surgical resection.

**Table 2:** Patterns of extragastric recurrence in early gastric cancer after curative resection.

Extragastric Recurrence (*n*, %)	16/1854 (0.9)
Mean time to recurrence (month ± SD)	25.7 ± 15.6
Method of resection (*n*, %)	
Endoscopic resection	4/782 (0.5)
Surgical resection	12/1072 (1.1)
Recurrence pattern (*n*, %)	
Endoscopic resection	
Regional lymph node	2/4 (50.0)
Liver	1/4 (25.0)
Peritoneum	1/4 (25.0)
Surgical resection	
Lung	3/12 (25.0)
Bone	3/12 (25.0)
Peritoneum	3/12 (25.0)
Regional lymph node	2/12 (16.7)
Liver	1/12 (8.3)
Distant lymph node	1/12 (8.3)
Adrenal gland	1/12 (8.3)

**Table 3:** Univariate and multivariate analyses of predictive factors for extragastric recurrence.

	Univariate Analysis			Multivariate Analysis	
Variable	No Recurrence (*n* = 1803)	Extragastric Recurrence (*n* = 16)	*p*-Value	OR (95% CI)	*p*-Value
Age (years, mean ± SD)	60.4 ± 11.9	58.5 ± 9.6	0.536		
Male (*n*, %)	1190 (66.0)	9 (56.3)	0.753		
Location (*n*, %)			0.010		
Upper third	159 (8.8)	4 (25.0)		1	
Middle third	422 (23.4)	7 (43.8)		0.620 (0.131–3.002)	0.602
Lower third	1222 (67.8)	5 (31.3)		0.290 (0.060–1.381)	0.174
Maximal diameter (mm, mean ± SD)	20.9 ± 17.0	22.8 ± 7.7	0.831		
Multiplicity (*n*, %)			0.081		
Single lesion	1733 (96.1)	15 (93.8)			
Multiple lesion	70 (3.9)	1 (6.3)			
Gross appearance (*n*, %)			0.675		
Elevated	356 (19.7)	4 (25.0)			
Flat	695 (38.5)	4 (25.0)			
Depressed	752 (41.7)	8 (50.0)			
Histologic type (*n*, %)			0.150		
Differentiated	1145 (63.5)	7 (43.9)			
Undifferentiated	658 (36.5)	9 (56.3)			
Lauren classification (*n*, %)			0.045		
Intestinal	1201 (66.6)	7 (43.8)		1	
Diffuse	510 (28.3)	6 (37.5)		1.253 (0.523–6.802)	0.517
Mixed	92 (5.1)	3 (18.8)		2.125 (0.412–11.331)	0.530
Depth of invasion (*n*, %)			<0.001		
Mucosa	1439 (79.8)	5 (31.3)		1	
Submucosa	364 (20.2)	11 (68.8)		6.351 (1.251–39.352)	0.032
Lymph node status * (*n*, %)			<0.001		
N0	970/1055 (91.9)	3/12 (25.0)		1	
N1	66/1055 (6.3)	1/12 (8.3)		2.412 (0.242–23.051)	0.452
N2	19/1055 (1.8)	3/12 (25.0)		2.710 (0.156–48.658)	0.495
N3	0 (0)	5/12 (41.7)		171.512 (4.359–7965.315)	0.012

* Only evaluated in the case of surgical resection.

## Data Availability

Not applicable.

## Supplementary Materials

The following supporting information can be downloaded at: https://www.mdpi.com/article/10.3390/jcm11113072/s1, Supplementary Table S1: Univariate analysis of lymph node status between the extragastric recurrence and the control groups.

## References

[1] Japanese Gastric Cancer Association (2011). Japanese gastric cancer treatment guidelines 2010 (ver. 3). Gastric Cancer.

[2] Espinel J., Pinedo E., Ojeda V., Del Rio M.G. (2015). Treatment modalities for early gastric cancer. World J. Gastrointest. Endosc..

[3] Suzuki H., Oda I., Abe S., Sekiguchi M., Mori G., Nonaka S., Yoshinaga S., Saito Y. (2016). High rate of 5-year survival among patients with early gastric cancer undergoing curative endoscopic submucosal dissection. Gastric Cancer.

[4] Katai H., Ishikawa T., Akazawa K., Isobe Y., Miyashiro I., Oda I., Tsujitani S., Ono H., Tanabe S., Fukagawa T. (2018). Five-year survival analysis of surgically resected gastric cancer cases in japan: A retrospective analysis of more than 100,000 patients from the nationwide registry of the japanese gastric cancer association (2001–2007). Gastric Cancer.

[5] Lee H.J., Kim Y.H., Kim W.H., Lee K.U., Choe K.J., Kim J.P., Yang H.K. (2003). Clinicopathological analysis for recurrence of early gastric cancer. Jpn. J. Clin. Oncol..

[6] Basili G., Nesi G., Barchielli A., Manetti A., Biliotti G. (2003). Pathologic features and long-term results in early gastric cancer: Report of 116 cases 8-13 years after surgery. World J. Surg..

[7] Kim Y.I., Kim Y.W., Choi I.J., Kim C.G., Lee J.Y., Cho S.J., Eom B.W., Yoon H.M., Ryu K.W., Kook M.C. (2015). Long-term survival after endoscopic resection versus surgery in early gastric cancers. Endoscopy.

[8] Lee S., Choi K.D., Hong S.M., Park S.H., Gong E.J., Na H.K., Ahn J.Y., Jung K.W., Lee J.H., Kim D.H. (2017). Pattern of extragastric recurrence and the role of abdominal computed tomography in surveillance after endoscopic resection of early gastric cancer: Korean experiences. Gastric Cancer.

[9] Seo N., Han K., Hyung W.J., Chung Y.E., Park C.H., Kim J.H., Lee S.K., Kim M.J., Noh S.H., Lim J.S. (2019). Stratification of postsurgical computed tomography surveillance based on the extragastric recurrence of early gastric cancer. Ann. Surg..

[10] Ricci A.D., Rizzo A., Rojas Llimpe F.L., Di Fabio F., De Biase D., Rihawi K. (2021). Novel HER2-Directed Treatments in Advanced Gastric Carcinoma: AnotHER Paradigm Shift?. Cancers.

[11] Rihawi K., Ricci A.D., Rizzo A., Brocchi S., Marasco G., Pastore L.V., Rojas Llimpe F.L., Golfieri R., Renzulli M. (2021). Tumor-Associated Macrophages and Inflammatory Microenvironment in Gastric Cancer: Novel Translational Implications. Int. J. Mol. Sci..

[12] Rizzo A., Mollica V., Ricci A.D., Maggio I., Massucci M., Rojas Llimpe F.L., Di Fabio F., Ardizzoni A. (2020). Third- and later-line treatment in advanced or metastatic gastric cancer: A systematic review and meta-analysis. Future Oncol..

[13] Zheng Z., Liu Y., Bu Z., Zhang L., Li Z., Du H., Ji J. (2014). Prognostic role of lymph node metastasis in early gastric cancer. Chin. J. Cancer Res..

[14] Suh D.D., Oh S.T., Yook J.H., Kim B.S., Kim B.S. (2017). Differences in the prognosis of early gastric cancer according to sex and age. Ther. Adv. Gastroenterol..

[15] Yokota T., Kunii Y., Teshima S., Yamada Y., Saito T., Takahashi M., Kikuchi S., Yamauchi H. (2000). Significant prognostic factors in patients with early gastric cancer. Int. Surg..

[16] Bausys R., Bausys A., Vysniauskaite I., Maneikis K., Klimas D., Luksta M., Strupas K., Stratilatovas E. (2017). Risk factors for lymph node metastasis in early gastric cancer patients: Report from eastern europe country-lithuania. BMC Surg..

[17] Cheong J.H., Yang H.K., Kim H., Kim W.H., Kim Y.W., Kook M.C., Park Y.K., Kim H.H., Lee H.S., Lee K.H. (2018). Predictive test for chemotherapy response in resectable gastric cancer: A multi-cohort, retrospective analysis. Lancet Oncol..

[18] Sasako M. (2020). Progress in the treatment of gastric cancer in japan over the last 50 years. Ann. Gastroenterol. Surg..

[19] Japanese Gastric Cancer Association (2017). Japanese gastric cancer treatment guidelines 2014 (ver. 4). Gastric Cancer.

[20] Marano L., D’Ignazio A., Cammillini F., Angotti R., Messina M., Marrelli D., Roviello F. (2019). Comparison between 7th and 8th edition of ajcc tnm staging system for gastric cancer: Old problems and new perspectives. Transl. Gastroenterol. Hepatol..

[21] Minata M., Audia A., Shi J., Lu S., Bernstock J., Pavlyukov M.S., Das A., Kim S.H., Shin Y.J., Lee Y. (2019). Phenotypic plasticity of invasive edge glioma stem-like cells in response to ionizing radiation. Cell Rep..

[22] Busuttil R.A., George J., House C.M., Lade S., Mitchell C., Di Costanzo N.S., Pattison S., Huang Y., Tan P., Cheong J. (2021). SFRP4 drives invasion in gastric cancer and is an early predictor of recurrence. Gastric Cancer.

[23] Choi Y.Y., Jang E., Kim H., Kim K.M., Noh S.H., Sohn T.S., Huh Y.M., An J.Y., Cheong J.H. (2021). Single patient classifier as a prognostic biomarker in pT1N1 gastric cancer: Results from two large Korean cohorts. Chin. J. Cancer Res..

[24] Sano T., Sasako M., Kinoshita T., Maruyama K. (1993). Recurrence of early gastric cancer. Follow-up of 1475 patients and review of the japanese literature. Cancer.

[25] Lai J.F., Kim S., Kim K., Li C., Oh S.J., Hyung W.J., Rha S.Y., Chung H.C., Choi S.H., Wang L.B. (2009). Prediction of recurrence of early gastric cancer after curative resection. Ann. Surg. Oncol..

[26] Youn H.G., An J.Y., Choi M.G., Noh J.H., Sohn T.S., Kim S. (2010). Recurrence after curative resection of early gastric cancer. Ann. Surg. Oncol..

[27] Huang K.H., Chen J.H., Wu C.W., Lo S.S., Hsieh M.C., Li A.F., Lui W.Y. (2009). Factors affecting recurrence in node-negative advanced gastric cancer. J. Gastroenterol. Hepatol..

[28] Choi H.J., Kim S.M., An J.Y., Choi M.G., Lee J.H., Sohn T.S., Bae J.M., Kim S. (2016). Risk factors and tumor recurrence in pt1n0m0 gastric cancer after surgical treatment. J. Gastric Cancer.

[29] Macalindong S.S., Kim K.H., Nam B.H., Ryu K.W., Kubo N., Kim J.Y., Eom B.W., Yoon H.M., Kook M.C., Choi I.J. (2018). Effect of total number of harvested lymph nodes on survival outcomes after curative resection for gastric adenocarcinoma: Findings from an eastern high-volume gastric cancer center. BMC Cancer.

[30] Liang C.J., Wang Z.W., Chang Y.W., Lee K.C., Lin W.H., Lee J.L. (2019). Sfrps are biphasic modulators of wnt-signaling-elicited cancer stem cell properties beyond extracellular control. Cell Rep..

[31] Deshmukh A., Arfuso F., Newsholme P., Dharmarajan A. (2018). Regulation of cancer stem cell metabolism by secreted frizzled-related protein 4 (sfrp4). Cancers.

[32] Lee J.L., Chang C.J., Chueh L.L., Lin C.T. (2006). Secreted frizzled related protein 2 (sfrp2) decreases susceptibility to uv-induced apoptosis in primary culture of canine mammary gland tumors by nf-kappab activation or jnk suppression. Breast Cancer Res. Treat..

[33] Chan C.W., Wong N.A., Liu Y., Bicknell D., Turley H., Hollins L., Miller C.J., Wilding J.L., Bodmer W.F. (2009). Gastrointestinal differentiation marker cytokeratin 20 is regulated by homeobox gene cdx1. Proc. Natl. Acad. Sci. USA.

[34] Almeida R., Silva E., Santos-Silva F., Silberg D.G., Wang J., De Bolos C., David L. (2003). Expression of intestine-specific transcription factors, cdx1 and cdx2, in intestinal metaplasia and gastric carcinomas. J. Pathol..

[35] Kang J.M., Lee B.H., Kim N., Lee H.S., Lee H.E., Park J.H., Kim J.S., Jung H.C., Song I.S. (2011). Cdx1 and cdx2 expression in intestinal metaplasia, dysplasia and gastric cancer. J. Korean Med. Sci..

[36] National Comprehensive Cancer Network (2017). Clinical Practice Guidelines in Oncology. Breast Cancer. Version 3. http://www.nccn.org/professionals/physician_gls/pdf/breast.pdf.

