# Supplementary material for: SFRP4 and CDX1 Are Predictive Genes for Extragastric Recurrence of Early Gastric Cancer after Curative Resection
Source: J Clin Med. 2022 May 29;11(11):3072. doi: 10.3390/jcm11113072 (PMC9181378; doi:10.3390/jcm11113072)
Supplement: Supplementary file 1 [file jcm-11-03072-s001.zip › jcm-1667461-supplementary.pdf]

Supplementary Table S1. Univariate analysis of lymph node status between the extragastric recurrence and the control groups.

**Variable**		**Control (*n* = 24)**	**Extragastric recurrence (*n* = 12)**	***p*-Value**
Lymph node status 1 (*n*, %)				1.000
	Negative	6/24 (25.0)	3/12 (25.0)	
	Positive	18/24 (75.0)	9/12 (75.0)	
Lymph node status 2 (*n*, %)				0.075
	N0	6/24 (25.0)	3/12 (25.0)	
	N1	6/24 (25.0)	1/12 (8.3)	
	N2	12/24 (50.0)	3/12 (25.0)	
	N3	0 (0)	5/12 (41.7)	
